# An optimal transition time to extracorporeal cardiopulmonary resuscitation for predicting good neurological outcome in patients with out-of-hospital cardiac arrest: a propensity-matched study

**DOI:** 10.1186/s13054-014-0535-8

**Published:** 2014-09-26

**Authors:** Su Jin Kim, Jae Seung Jung, Jae Hyoung Park, Jong Su Park, Yun Sik Hong, Sung Woo Lee

**Affiliations:** Department of Emergency Medicine, College of Medicine, Korea University, Inchon-ro 73, Seongbuk-gu, Seoul, 136-705 Korea; Department of Thoracic and Cardiovascular Surgery, College of Medicine, Korea University, Seoul, Korea; Department of Internal Medicine, Subdivision of Cardiovascular Medicine, College of Medicine, Korea University, Seoul, Korea

## Abstract

**Introduction:**

Prolonged conventional cardiopulmonary resuscitation (CCPR) is associated with a poor prognosis in out-of-hospital cardiac arrest (OHCA) patients. Alternative methods can be needed to improve the outcome in patients with prolonged CCPR and extracorporeal cardiopulmonary resuscitation (ECPR) can be considered as an alternative method. The objectives of this study were to estimate the optimal duration of CPR to consider ECPR as an alternative resuscitation method in patients with CCPR, and to find the indications for predicting good neurologic outcome in OHCA patients who received ECPR.

**Methods:**

This study is a retrospective analysis based on a prospective cohort. We included patients ≥ 18 years of age without suspected or confirmed trauma and who experienced an OHCA from May 2006 to December 2013. First, we determined the appropriate cut-off duration for CPR based on the discrimination of good and poor neurological outcomes in the patients who received only CCPR, and then we compared the outcome between the CCPR group and ECPR group by using propensity score matching. Second, we compared CPR related data according to the neurologic outcome in matched ECPR group.

**Results:**

Of 499 patients suitable for inclusion, 444 and 55 patients were enrolled in the CCPR and ECPR group, respectively. The predicted duration for a favorable neurologic outcome (CPC1, 2) is < 21 minutes of CPR in only CCPR patients. The matched ECPR group with ≥ 21 minutes of CPR duration had a more favorable neurological outcome than the matched CCPR group at 3 months post-arrest. In matched ECPR group, younger age, witnessed arrest without initial asystole rhythm, early achievement of mean arterial pressure ≥ 60 mmHg, low rate of ECPR-related complications, and therapeutic hypothermia were significant factors for expecting good neurologic outcome.

**Conclusions:**

ECPR should be considered as an alternative method for attaining good neurological outcomes in OHCA patients who required prolonged CPR, especially of ≥ 21 minutes. Younger or witnessed arrest patients without initial asystole were good candidates for ECPR. After implantation of ECPR, early hemodynamic stabilization, prevention of ECPR-related complications, and application of therapeutic hypothermia may improve the neurological outcome.

## Introduction

The survival rate to discharge of out-of-hospital cardiac arrest (OHCA) patients remains poor, despite decades of advanced practice in conventional cardiopulmonary resuscitation (CCPR) [1-5]. The causes of high mortality in OHCA are the failure to establish a return of spontaneous circulation (ROSC) and multiple organ failure, including hypoxic brain injury in patients receiving CCPR [6]. The rate of favorable neurological outcomes in OHCA patients decreases according to the prolongation of CPR duration [1]. Therefore, alternative resuscitative methods may be necessary to improve survival, especially in patients who require prolonged CPR [7].

The American Heart Association (AHA) recommended the classification of extracorporeal cardiopulmonary resuscitation (ECPR) as an alternative method for patients who have a brief no-flow time and a reversible cause of cardiac arrest as Class IIb [8,9]. ECPR used as treatment of cardiac arrest may preserve myocardial viability by enhancing coronary blood flow, thus increasing the chance of ROSC [10]. As ECPR provides sufficient perfusion to vital organs such as the brain and the injured myocardium, the window of the duration of effective resuscitation could be extended when using ECPR [11]. In addition, ECPR may increase long-term survival by ensuring adequate oxygenated blood delivery to end organs until an effective cardiac output has been recovered, thus preventing organ failure.

There are few studies on the optimal time to consider alternative resuscitation methods in OHCA patients who receive prolonged CPR. Moreover, the advantage of ECPR as an alternative method for attaining a good cerebral performance category (CPC) score in OHCA patients compared to CCPR is not well known.

We hypothesized that alternative methods may be considered after the optimal cutoff time due to the decreasing rate of good neurological outcomes according to a prolonged duration of CPR. Additionally, after the estimated cutoff time, an effective alternative method, such as ECPR compared to CCPR, may show better neurological outcomes. The primary goal of this study was to estimate the optimal duration of CPR to consider ECPR as an alternative resuscitation method in patients with CCPR, and to determine whether ECPR results in better neurological outcomes than CCPR in patients who require prolonged CPR beyond the optimal duration in OHCA patients. The second goal was to find the indications for predicting good neurologic outcome in patients who receive ECLS.

## Materials and methods

### Design and setting

This study was a retrospective analysis based on a prospective cohort study that was conducted at the emergency department (ED) of the Korea University Medical Center (KUMC), comprising 44 beds of the 890-bed university teaching hospital, from May 2006 to December 2013. We analyzed the CPR registry that comprised prospectively collected data on pre- and in-hospital variables of cardiac arrest patients who received CPR. The Institutional Review Board (IRB) of KUMC approved the data collection for the establishment of the CPR cohort and informed consent for the CPR cohort and for ECPR was also obtained prior to or after the institution of ECPR, from the families of all cardiac arrest patients, respectively. The IRB of KUMC approved this retrospective analysis separately from establishment of the cohort.

### Data collection of the CPR registry

A CPR coordinator prospectively collected data for the CPR registry according to the Utstein style guideline [12]. The registry included demographic data; comorbidities; whether the cardiac arrest was witnessed; the incidence of suspected or confirmed trauma; presumed time of cardiac arrest; presence of bystander CPR; first documented cardiac arrest rhythm by the emergency medical service (EMS) provider; any return of spontaneous circulation (ROSC); presence of ECPR; the presence of return of spontaneous heart beating (ROSB) after ECPR; presumed cause of cardiac arrest; application of therapeutic hypothermia, coronary angiography (CAG), or percutaneous coronary intervention (PCI); 24-hour survival; the presence of ROSC for ≥20 minutes, hospital length of stay (LOS) in survivors at discharge; CPC score at discharge and 3 months post cardiac arrest; and the final diagnosis at discharge. The CPC score at 3-months post cardiac arrest was obtained from outpatient clinic medical record review and telephone interviews conducted by the CPR coordinator.

The comorbidity score was calculated using the Charlson comorbidity index [13]. The time intervals were calculated from the registry. The interval between cardiac arrest and start of CPR was defined as the time from when the arrest was witnessed or found, to CPR started by EMS providers, and the CPR duration was defined as the time interval from the first chest compression provided by EMS providers to the termination of resuscitation efforts either because of ROSC (for ≥20 minutes), implantation of ECPR, or declaration of death [14]. If an OHCA patient had recurrent cardiac arrest after gaining ROSC (for ≥20 minutes), the first ROSC (≥20 minutes) time was defined as the CPR stop-time. The CPR duration consisted of pre-hospital CPR duration (CPR start to ED presentation) and in-hospital CPR duration (ED presentation to CPR stop).

### Indications and management of ECPR in the KUMC ED

The indications for ECPR in the KUMC ED during the study period were 1) age ≥18 years; 2) sudden cardiac arrest with presumed correctable causes; 3) witnessed cardiac arrest with or without bystander CPR; or 4) no-flow time (time interval from presumed arrest to CPR started by the EMS provider) was expected to be short, even for unwitnessed arrests. The initial cardiac arrest rhythm documented pre-hospitalization was not considered as an indication for ECPR. The contraindications for ECPR were 1) cardiac arrest due to a clearly uncorrectable cause; 2) presence of a terminal illness or malignancy; 3) suspected or confirmed traumatic origin of arrest; and 4) no informed consent from the family. The ECPR team was activated by the emergency physician in cases when OHCA patients met the inclusion criteria, and required prolonged CPR more than 10 minutes as in-hospital CPR duration or recurrently arrested in the ED after achievement of ROSC (≥20 minutes). The time from activating the ECPR team to implantation of ECPR was 10 to 15 minutes during the day and 20 to 25 minutes during the night in our institution. ECPR was implemented in the ED or coronary catheterization room.

All patients who experienced cardiac arrest received advanced cardiac life support by emergency physicians according to the AHA guidelines, excluding patients with a do not attempt resuscitation (DNAR) order or irreversible signs of death. The ECPR team consisted of emergency physicians, cardiovascular surgeons, coronary intervention specialists, and perfusionists. A twin-pulse extracorporeal life support system (T-PLS®: NewHeartbio, Seoul, Korea) or a Capiox emergency bypass system (EBS®; Terumo Inc., Tokyo, Japan) were used for ECPR. Depending on the patient’s body size, a 15- to 17-Fr arterial and a 21 to 23-Fr venous catheter were inserted into the femoral artery and vein percutaneously using Seldinger’s technique while maintaining chest compressions. The flow rate was initially set at 2.5 to 3.0 L/min. Anticoagulation with heparin was given immediately after initiation of ECLS and titrated to maintain an activated clotting time of 200 to 220 seconds.

Intra-arterial blood pressure and arterial blood gas analysis at the radial artery were monitored. An intra-aortic balloon pump was not used in the ED. After implementation of ECPR, CAG was performed as soon as possible in cases of suspected acute coronary syndrome (ACS).

ECPR was discontinued if spontaneous heart beating was not obtained despite correction of the cause of cardiac arrest. Withdrawal of ECPR was considered if there was evidence of multiple organ failure, refractory shock, or irreversible neurologic injury and with consent from the patient’s family. A weaning protocol was instituted after assessing hemodynamic profiles and myocardial functions by echocardiography while progressively reducing extracorporeal membrane oxygenation (ECMO) flow of 1.5 L/min [15,16].

### Selection of study patients and outcome measurements

**Figure 1:**
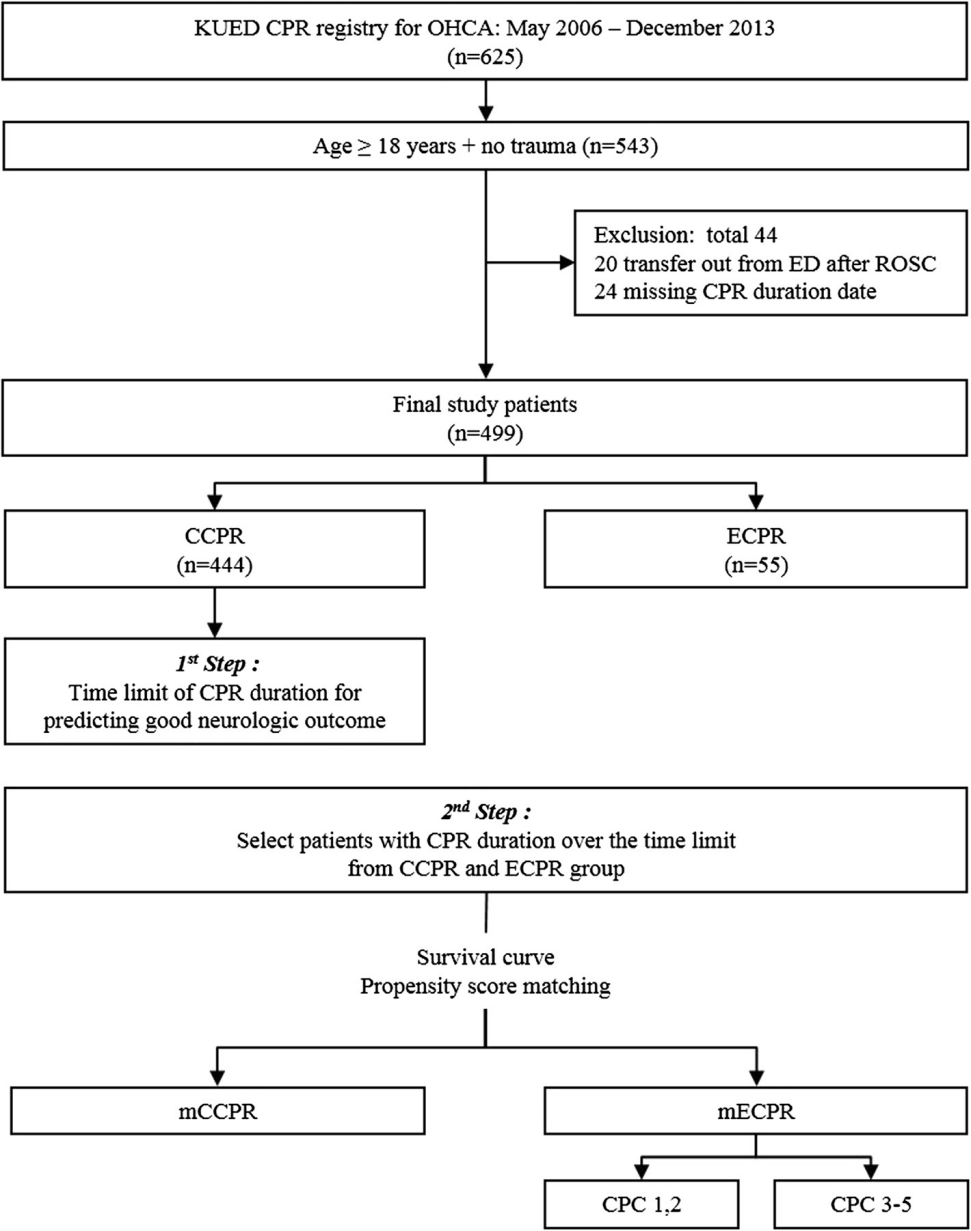
**Selection of study patients and study design.** KUED, Korea university emergency department; OHCA, out-of-hospital cardiac arrest; DNAR, do not attempt resuscitation; CPR, cardiopulmonary resuscitation; ECPR, extracorporeal CPR group; CCPR, conventional CPR group; mECPR, matched ECPR group; mCCPR, matched CCPR group; CPC, cerebral performance category.

We enrolled the patients aged 18 years or older who experienced OHCA, with no traumatic origin of cardiac arrest, in the study from the CPR registry cohort. The patients who were transferred from the ED to other hospitals after ROSC and those who had missed the CPR duration date were excluded (Figure 1). The primary end point was a good neurological outcome (measured as a CPC score of 1 or 2) at 3 months post cardiac arrest [17]. The secondary end points were 24-hour survival rate, survival to discharge, and survival rate at 3 months post arrest.

### Data analysis

Receiver operating characteristic (ROC) curve analysis of CPR duration was used to differentiate between favorable and unfavorable neurological outcomes in the CCPR group. We compared good neurological outcomes by using a Kaplan-Meier plot for ECPR and CCPR groups with CPR duration longer than the estimated cut off duration. A propensity-score-matching analysis was performed to reduce the effects of selection bias and possible confounding factors. The propensity score, which was the predicted probability of receiving ECPR with covariates, was derived through a logistic regression model. The covariates, including age, sex, comorbidity score, bystander CPR, witnessed cardiac arrest, first documented arrest rhythm, presumed etiology of arrest, interval from arrest to CPR started by EMS provider, CPR duration, and therapeutic hypothermia, were used in propensity-score-matching. Immediate CAG for only ACS suspected patients was not matched at propensity-matching analysis, because our patients had variable causes of cardiac arrest. ECPR and CCPR cases were matched by their propensity score in blocks of 1:1 and the selected patients formed well-matched 1:1 pairs in both groups [15,16]. Model discrimination was assessed with *C* statistics. We compared the characteristics according to the good neurologic outcome to find prognostic factors in matched ECPR group.

The data were reported as median with interquartile range (IQR). Continuous variables were compared using the Mann-Whitney test. Categorical variables were compared using the chi-square or Fisher’s exact test. All statistical analyses were carried out using SPSS version 20.0 (IBMSPSS Inc., Chicago, IL, USA) with the propensity-score-matching program. Two-tailed *P*-values f <0.05 were considered statistically significant.

## Results

### Characteristics, CPR variables and outcomes of the unmatched ECPR and CCPR group

A total of 625 patients who received CPR due to OHCA at ED were registered with the CPR registry during the study period, of which 543 patients were enrolled in this study. Of these, 499 patients were included in our study group according to the selection criteria; 444 were included in the CCPR group and 55 in ECPR group (Figure 1). The baseline characteristics and CPR variables of both groups are shown in Table 1.

**Table 1:** **Comparison of characteristics between the ECPR group and CCPR group**

	**ECPR (n = 55)**	**CCPR (n = 444)**	***P*** **-value**
Age, years	53 (41 to 68)	69 (56 to 77)	<0.001
Male : female, n	41 : 14	285 : 159	0.136
Pre-existing comorbidity, n (%)			
Hypertension	14 (25.5)	161 (36.3)	0.134
Cardiovascular disease	15 (27.3)	101 (22.7)	0.498
Chronic pulmonary disease	1 (1.8)	33 (7.4)	0.157
Neurologic disease	3 (5.5)	67 (15.1)	0.062
Chronic liver disease	1 (1.8)	20 (4.5)	0.495
Chronic kidney disease	1 (1.8)	41 (9.2)	0.070
Diabetes	11 (20.0)	122 (27.5)	0.262
Malignancy	1 (1.8)	38 (8.6)	0.106
Number of pre-existing comorbidities	0 (0 to 1)	1 (0 to 2)	0.003
Comorbidity score	0 (0 to 1)	1 (0 to 2)	<0.001
Witnessed, n (%)	43 (78.2)	328 (73.9)	0.623
Bystander CPR, n (%)	23 (41.8)	151 (34.0)	0.294
First documented arrest rhythm, n (%)			<0.001
VF/VT	31 (56.4)	85 (19.1)	
PEA	10 (18.2)	91 (20.5)	
Asystole	14 (25.5)	268 (60.4)	
Arrest to CPR start, minutes	7 (0 to 13)	8 (5 to 12)	0.108
CPR duration, minutes	62 (47 to 89)	35 (21 to 50)	<0.001
Pre-hospital CPR duration	13 (7 to 17)	13 (8 to 17)	0.930
In-hospital CPR duration	47 (35 to 80)	21 (8 to 35)	<0.001
Presumed etiology of arrest, n (%)			<0.001
Cardiac	49 (89.1)	277 (62.4)	
Non-cardiac	6 (10.9)	167 (37.6)	
At the time of admission to ED			
SAPS II*	91 (87 to 97)	97 (91 to 103)	<0.001
Arterial pH*	6.98 (6.86 to 7.05)	6.94 (6.83 to 7.06)	0.473
Serum lactate*	11.7 (8.8 to 16.0)	10.8 (7.3 to 14.0)	0.083
Any ROSC events during CPR, n (%)	17 (30.9)	94 (21.2)	0.121
Therapeutic hypothermia, n (%)	17 (30.9)	71 (16.0)	0.013
ROSB/ROSC, n (%)	44 (80.0)	212 (47.7)	<0.001
Survival to 24 hours, n (%)	32 (58.2)	138 (31.1)	<0.001
CPC score at discharge			0.226
1, n (%)	7 (12.1)	30 (6.8)	
2, n (%)	1 (1.8)	6 (1.4)	
3, n (%)	0 (0)	8 (1.8)	
4, n (%)	1 (1.8)	42 (9.5)	
5, n (%)	46 (83.6)	358 (80.6)	
Hospital LOS in survival to discharge, day	30 (14 to 60)	18 (9 to 30)	0.120
Survivor at 3 months after arrest, n (%)	8 (14.5)	44 (9.9)	0.346
CPC 1,2 at 3 months after arrest, n (%)	8 (14.5)	36 (8.1)	0.128

Data are shown as median (interquartile range) or number (%). *Measured in 48 ECPR patients and 332 CCPR patients. CPR, cardiopulmonary resuscitation; ECPR, extracorporeal CPR; CCPR, conventional CPR; VF, ventricular fibrillation; VT, ventricular tachycardia; PEA, pulseless electrical activity; ED, emergency department; SAPS, simplified acute physiologic score; ROSB, return of spontaneous heart beat; ROSC, return of spontaneous circulation; CPC, cerebral performance category; LOS, length of stay.

Patients in the ECPR group had significantly younger age, lower comorbidity score, and higher incidence of shockable rhythm as the first documented arrest rhythm than those in the CCPR group. The CPR duration and the incidence of presumed etiology of cardiac arrest was longer and higher in the ECPR than in the CCPR group (Table 1). A good neurological outcome at 3 months post arrest did not show any significant difference between groups (Table 1).

### The trend of outcomes according to the CPR duration in both unmatched groups

**Figure 2:**
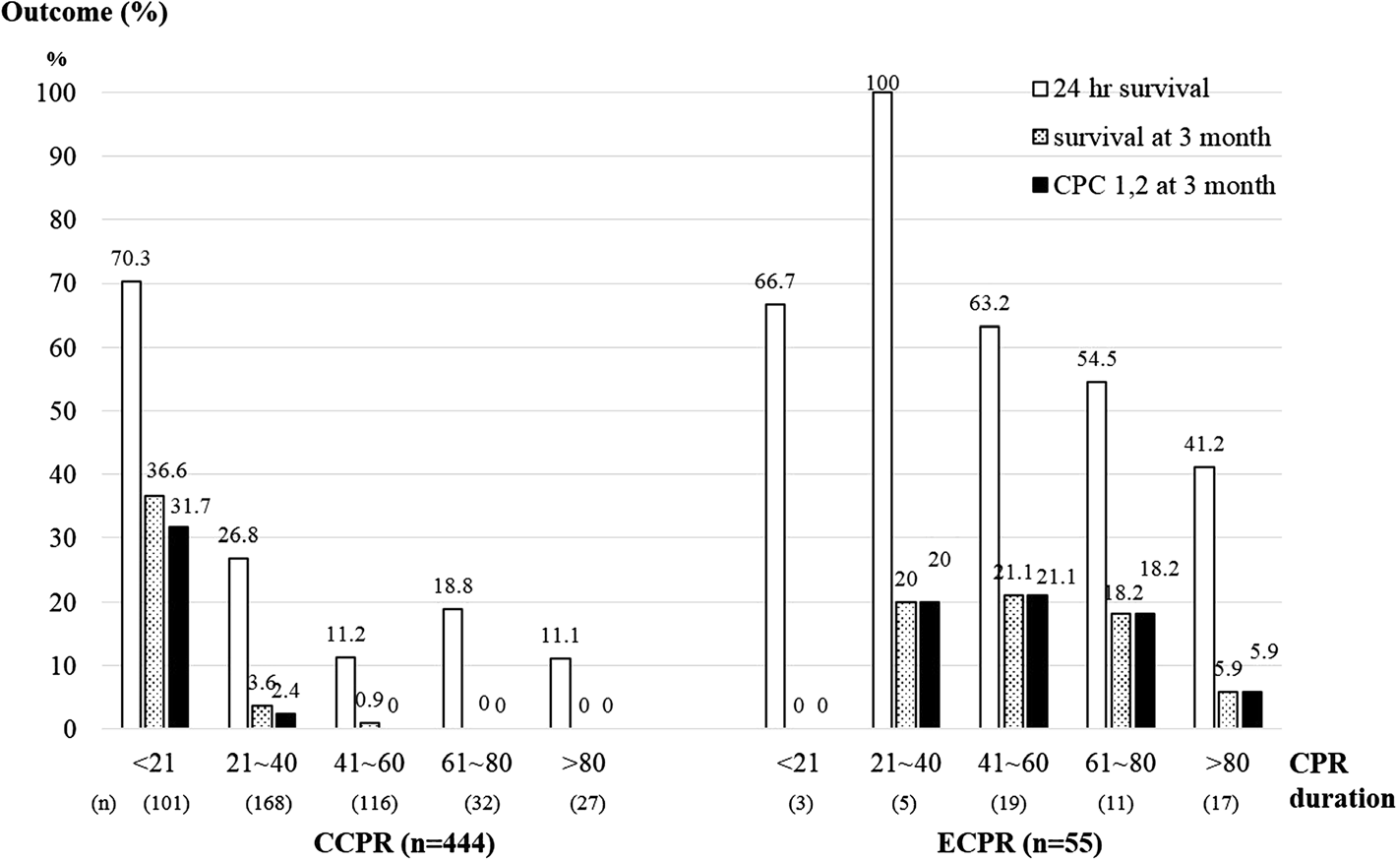
**Trends of outcomes in the conventional cardiopulmonary resuscitation (CCPR) and extracorporeal cardiopulmonary resuscitation (ECPR) groups according to the cardiopulmonary resuscitation (CPR) duration.** In the ECPR group, the longest CPR duration with a good neurologic outcome was 120 minutes. CPC, cerebral performance category.

The rates of ROSC or ROSB, 24-hour survival, survival rate at 3 months post cardiac arrest, and good neurological outcome (CPC 1, 2) at 3 months post arrest tended to decrease more sharply in the CCPR than in the ECPR group according to the prolongation of CPR duration (Figure 2).

### ROC curve of CPR duration to predict good neurologic outcome in the CCPR group

**Figure 3:**
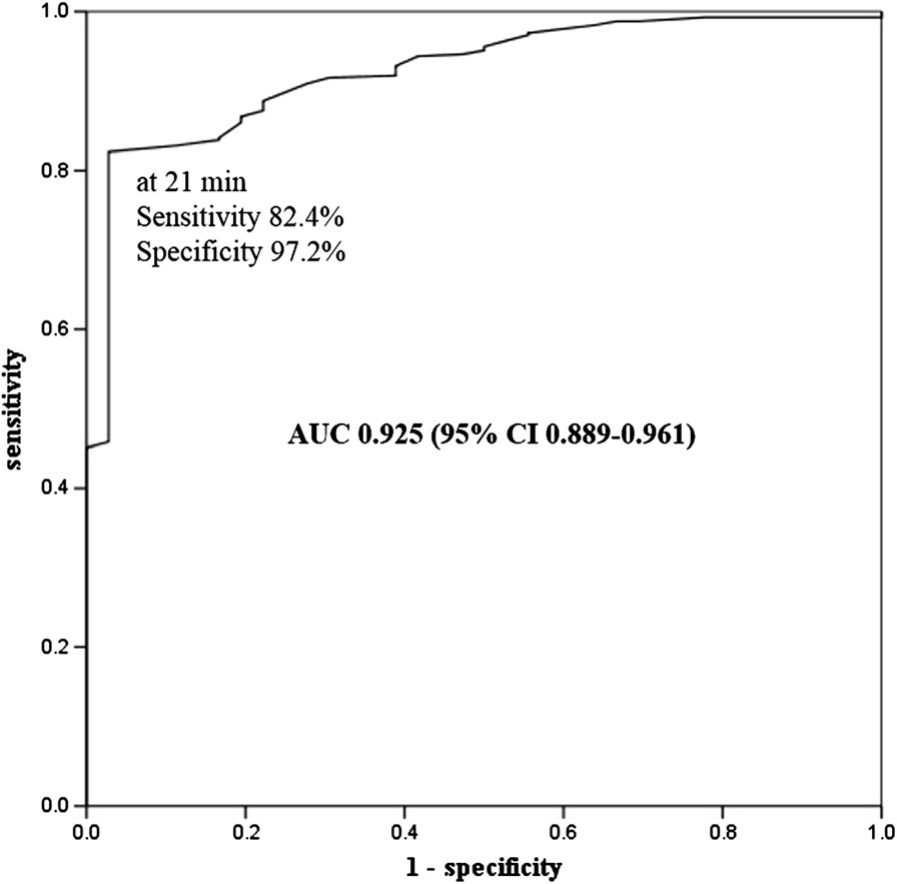
**Receiver-operating characteristic (ROC) curves for the cutoff time of cardiopulmonary resuscitation (CPR) duration for predicting a good neurological outcome in the conventional cardiopulmonary resuscitation (CCPR) group (** ***P*** **<0.001).** The cutoff value was 21 minutes. AUC, area under the curve.

Using a ROC-curve analysis of the CCPR group (n = 444), the best discriminative CPR duration for a favorable neurological outcome was 21 minutes (area under the curve 0.925, 95% CI 0.889, 0.961, *P* <0.001) (Figure 3).

### Comparison of ECPR and CCPR group survival analysis in patients with CPR duration ≥21 minutes

**Figure 4:**
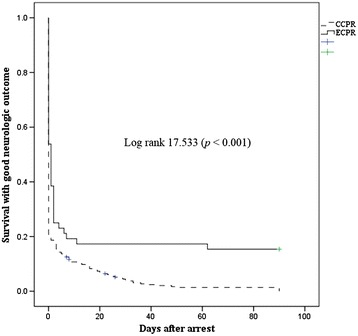
**Kaplan-Meier plot of survival with a good neurological outcome at 3 months post cardiac arrest for patients who experienced out-of-hospital cardiac arrest** **(OHCA) with cardiopulmonary resuscitation (CPR) duration ≥21 minutes.** The extracorporeal CPR (ECPR) group showed better neurological outcomes compared to the conventional CPR (CCPR) group at 3 monthS post arrest.

Survival analysis using a Kaplan-Meier plot showed more favorable neurological outcomes in the ECPR than in the CCPR group at 3 months post cardiac arrest among patients with a CPR duration ≥21 minutes (stratified log-rank test, *P* <0.001) (Figure 4).

### Outcome analysis of propensity-score-matched groups (in cases of CPR duration ≥21 minutes)

**Figure 5:**
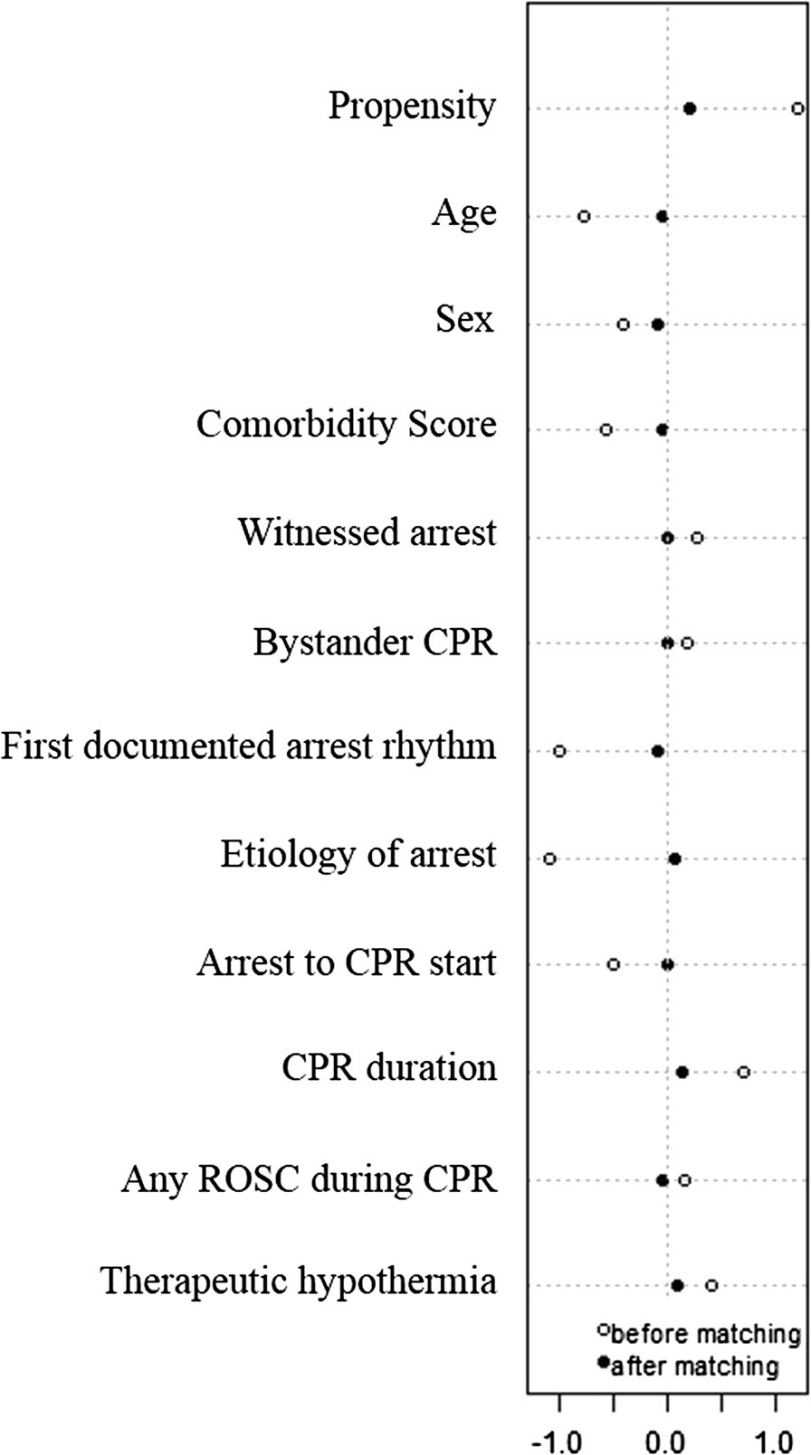
**Dot-plot of standardized mean differences before and after matching in study patients with a cardiopulmonary resuscitation (CPR) duration ≥21 minutes.** ROSC = return of spontaneous circulation.

**Figure 6:**
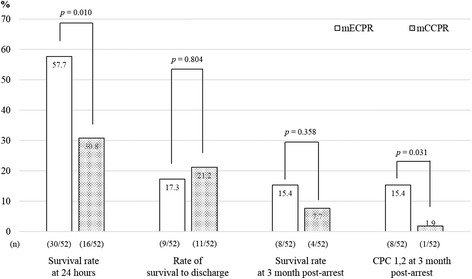
**Outcomes of the matched extracorporeal cardiopulmonary resuscitation (mECPR) and matched conventional cardiopulmonary resuscitation (mCCPR) groups after propensity-score-matching in patients with cardiopulmonary resuscitation (CPR) duration ≥21 minutes.** CPC, cerebral performance category.

The *c*-statistic of the propensity-score model was 0.999, and the median propensity scores in the ECPR and CCPR groups were 0.39 (SD 0.14) versus 0.10 (SD 0.14), respectively, before matching and 0.39 (SD 0.20) versus 0.34 (SD 0.20), respectively, after matching (Figure 5). The confounding factors of the characteristics were balanced and there were no significant differences in the matched groups (Table 2). The median value of CPR duration was 63 minutes (IQR 50 to 88) and 61 minutes (IQR 40 to 84) in the matched ECPR group (mECPR) and the matched CCPR group (mCCPR), respectively. Application of therapeutic hypothermia (TH) was not different between mECPR and mCCPR (*P* = 0.821). CAG for suspected acute coronary disease was more frequent in mECPR than mCCPR (*P* = 0.213) (Table 3). The rate of 24-hour survival and a favorable neurological outcome at 3 months post cardiac arrest in the mECPR group were significantly higher than those in the mCCPR group, although the rate of survival to discharge was similar in both groups (Figure 6).

**Table 2:** **Comparison of the characteristics between the mECPR and mCCPR groups**

	**mECPR (n = 52)**	**mCCPR (n = 52)**	***P*** **-value**
Age, years	54 (41 to 69)	54 (42 to 68)	0.871
Male : female, n	40 : 12	38 : 14	0.821
Pre-existing comorbidity, n (%)			
Hypertension	13 (25.0)	12 (23.1)	1.000
Cardiovascular disease	15 (28.8)	11 (21.2)	0.497
Chronic pulmonary disease	1 (1.9)	0 (0.0)	1.000
Neurologic disease	3 (5.8)	4 (7.7)	1.000
Chronic liver disease	1 (1.9)	2 (3.8)	1.000
Chronic kidney disease	1 (1.9)	3 (5.8)	0.618
Diabetes	11 (21.2)	6 (11.5)	0.289
Malignancy	1 (1.9)	2 (3.8)	1.000
Number of pre-existing comorbidities	1 (0 to 2)	1 (0 to 1)	0.695
Comorbidity score	0 (0 to 1)	0 (0 to 1)	0.692
Witnessed, n (%)	42 (80.8)	42 (80.8)	1.000
Bystander CPR, n (%)	22 (42.3)	22 (30.8)	1.000
First documented arrest rhythm, n (%)			0.639
VF/VT	31 (59.6)	29 (55.8)	0.901
PEA	8 (15.4)	8 (15.4)	
Asystole	13 (25.0)	15 (28.8)	
Arrest to CPR start, minutes	7.0 (0.3 to 12.8)	7.0 (5.0 to 10.0)	0.507
CPR duration, minutes	62.5 (49.0 to 88.0)	60.5 (40.0 to 83.5)	0.387
Pre-hospital CPR duration	13.0 (7.0 to 17.0)	13.0 (9.3 to 16.8)	0.677
In-hospital CPR duration	47.5 (35.3 to 76.8)	48.0 (25.3 to 71.0)	0.550
Presumed etiology of arrest, n (%)			1.000
Cardiac	49 (94.2)	49 (94.2)	
Suspected ACS	44 (84.6)	46 (88.5)	
Non-cardiac	3 (5.8)	3 (5.8)	
At the time of admission to ED			
SAPS II*	91 (87 to 98)	91 (88 to 96)	0.695
Arterial pH*	6.98 (6.86 to 7.05)	6.97 (6.90 to 7.05)	0.487
Serum lactate*	11.6 (8.6 to 15.3)	10.8 (8.8 to 15.0)	0.460
Any ROSC events during CPR, n (%)	16 (30.8)	17 (32.7)	1.000

Data are shown as median (IQR) or number (%). *Measured in 48 mECPR and 47 mCCPR patients. CPR, cardiopulmonary resuscitation; ECPR, extracorporeal CPR; CCPR, conventional CPR; VF, ventricular fibrillation; VT, ventricular tachycardia; PEA, pulseless electrical activity; ED, emergency department; ACS, acute coronary syndrome; SAPS, simplified acute physiologic score; ROSC, return of spontaneous circulation.

**Table 3:** **Comparison of post-resuscitation care and outcomes between the mECPR and mCCPR groups**

	**mECPR (n = 52)**	**mCCPR (n = 52)**	***P*** **-value**
Rate of ROSB/ROSC (≥20 minutes), n (%)	42 (80.8)	20 (38.5)	< 0.001
Therapeutic hypothermia, n (%)	14 (26.9)	12 (23.1)	0.821
CAG in patients with suspected ACS (%)	39 in 44 (88.6)	11 in 15 (73.3)*	0.213
CAG findings	n = 39	n = 11	0.010
No significant coronary lesion	5 (12.8)	4 (36.4)	
Coronary spasm	3 (7.7)	4 (36.4)	
Presence of coronary occlusion/stenosis, n (%)	31 (79.5)	3 (27.3)	
Diseased coronary artery, n (%)	n = 31	n = 3	0.603
1 vessel	23 (74.2)	3 (100)	
2 vessels	5 (16.1)	0 (0)	
3 vessels	3 (9.7)	0 (0)	
PCI for coronary occlusion/stenosis	29 (93.5)	3 (100)	1.000
CPC score at discharge			0.011
1, n (%)	7 (13.5)	1 (1.9)	
2, n (%)	1 (1.9)	0	
3, n (%)	0	2 (3.8)	
4, n (%)	1 (1.9)	8 (15.4)	
5, n (%)	43 (82.7)	41 (78.8)	
Hospital LOS in survival to discharge, days	30 (14 to 60)	28 (16 to 50)	0.766
CPC score at 3 months post arrest			0.070
1, n (%)	7 (13.5)	1 (1.9)	
2, n (%)	1 (1.9)	0 (0)	
3, n (%)	0 (0)	2 (3.8)	
4, n (%)	0 (0)	1 (1.9)	
5, n (%)	44 (84.6)	48 (92.3)	
Final diagnosis of arrest, n (%)			< 0.001
Cardiac			
ACS/AMI	36 (69.2)	9 (17.3)	
Lethal arrhythmia	3 (5.8)	5 (9.6)	
HF, HCMP, DCMP	3 (5.8)	2 (3.8)	
Pulmonary embolism	2 (3.8)	1 (1.9)	
Unknown sudden arrest	4 (7.7)	31 (59.6)	
Non-cardiac			
Respiratory arrest	1 (1.9)	0 (0)	
Hypothermia	1 (1.9)	0 (0)	
Acute kidney injury	1 (1.9)	3 (5.8)	
Brain hemorrhage	1 (1.9)	1 (1.9)	
Causes of death at 3 months post arrest, n (%)	n = 44	n = 48	< 0.001
No ROSB or ROSC	10 (22.7)	32 (66.7)	
Refractory shock	24 (54.5)	4 (8.4)	
Hypoxic brain injury and organ failure	8 (18.2)	12 (25.0)	
Brain death	2 (4.5)	0 (0)	

Data are shown as median (IQR) or number (%). *In 15 suspected ACS patients with ROSC (≥20 minutes). CPR, cardiopulmonary resuscitation; ECPR, extracorporeal CPR; CCPR, conventional CPR; ROSB, return of spontaneous heart beat; ROSC, return of spontaneous circulation; CAG, coronary angiography; ACS, acute coronary syndrome; PCI, percutaneous coronary intervention; CPC, cerebral performance category; LOS, length of stay; HF, heart failure; HCMP, hypertrophic cardiomyopathy; DCMP, dilated cardiomyopathy.

### Comparison of CPR data according to the neurologic outcome in the mECPR group

Of 52 mECPR patients, 8 patients received ECPR due to recurrent cardiac arrest following ROSC (≥20 minutes). The median CPR duration and median time interval from ROSC to ECPR implantation was 68.5 (range 32 to 136) hours and 1.5 (range 0.6 to 6.4) hours, respectively. Younger age and witnessed arrest without initial asystole were significant indicators for predicting good neurologic outcome in the mECPR group (Table 3). The CPR duration of the group with good neurologic outcome tended to be short compared with those with poor neurologic outcome (*P* = 0.432). The rates of mean arterial pressure (MAP) ≥60 mmHg within 2 hours post ECPR and the application of therapeutic hypothermia were higher, and the rates of ECPR-related complications were lower in the group with CPC 1 and 2 than in the group with CPC 3 to 5 (Table 4). Inotropic equivalent dose was higher in the group with CPC 3 to 5 than in the group with CPC 1 and 2 (Table 4) [18]. The rate of the patients without any coronary lesions was higher in the group with good neurologic outcome. (Table 5).

**Table 4:** **Comparison of the baseline characteristics according to good neurologic outcome in the mECPR group (n = 52)**

	**CPC 1, 2 (n = 8)**	**CPC 3 to 5 (n = 44)**	***P*** **-value**
Age, years	37 (22 to 54)	56 (43 to 70)	0.014
Male : female, n	7 : 1	33 : 11	0.663
Pre-existing comorbidity, n (%)			
Hypertension	0 (0)	13 (29.5)	0.177
Cardiovascular disease	2 (25.0)	13 (29.5)	1.000
Chronic pulmonary disease	0 (0)	1 (2.3)	1.000
Neurologic disease	0 (0)	1 (2.3)	1.000
Chronic liver disease	0 (0)	1 (2.3)	1.000
Chronic kidney disease	0 (0)	1 (2.3)	1.000
Diabetes	1 (12.5)	10 (22.7)	1.000
Malignancy	1 (12.5)	0 (0)	0.154
Number of pre-existing comorbidities	0 (0 to 1)	1 (0 to 2)	0.135
Comorbidity score	0 (0 to 1)	0 (0 to 1)	0.367
Witnessed, n (%)	8 (100)	34 (77.3)	0.328
Bystander CPR, n (%)	5 (62.5)	17 (38.6)	0.260
First documented arrest rhythm, n (%)			0.191
VF/VT	6 (75.0)	25 (56.8)	
PEA	2 (25.0)	6 (13.6)	
Asystole	0 (0)	13 (29.5)	
Arrest to CPR start, minutes	6.0 (1.0 to 13.8)	7.0 (0.3 to 12.8)	0.794
CPR duration, minutes	57.0 (52.3 to 65.8)	67 (49 to 94)	0.432
Pre-hospital duration	14.0 (11.0 to 19.0)	13.0 (6.0 to 17.0)	0.525
In-hospital duration	38.5 (31.8 to 51.0)	51.0 (37.0 to 82.5)	0.233
Any ROSC events during CPR	4 (50.0)	12 (27.3)	0.231
At the time of admission to ED			
SAPS II^*^	87 (85 to 94)	91 (88 to 98)	0.140
arterial pH^*^	6.90 (6.81 to 7.04)	6.98 (6.86 to 7.05)	0.373
Serum lactate^*^	11.2 (9.3 to 16.5)	11.5 (8.5 to 14.9)	0.766
Reason for ECPR			0.593
Refractory arrest, n (%)	6 (75.0)	38 (86.4)	
Recurrent arrest, n (%)	2 (25.0)	6 (13.6)	
Location of ECPR implantation			
ED : catheterization room, n	2 : 6	18 : 26	0.463
Final diagnosis of arrest, n (%)			0.191
Cardiac	8 (100)	40 (90.3)	
ACS/AMI	5 (62.5)	31 (70.5)	
Lethal arrhythmia	2 (25.0)	1 (2.3)	
HF, HCMP, DCMP	0 (0)	3 (6.8)	
Pulmonary embolism	1 (12.5)	1 (2.3)	
Unknown sudden arrest	0 (0)	4 (9.1)	
Non-cardiac	0 (0)	4 (9.1)	
Respiratory arrest	0 (0)	1 (2.3)	
Hypothermia	0 (0)	1 (2.3)	
Acute kidney injury	0 (0)	1 (2.3)	
Brain hemorrhage	0 (0)	1 (2.3)	
Witnessed + no asystole	8 (26.7)	0 (0)	0.015

Data are shown as median (IQR) or number (%). *Measured in eight patients with CPC 1, 2 and 40 patients with CPC 3 to 5. CPR, cardiopulmonary resuscitation; ECPR, extracorporeal CPR; VF, ventricular fibrillation; VT, ventricular tachycardia; PEA, pulseless electrical activity; ED, emergency department; SAPS, simplified acute physiologic score; ROSC, return of spontaneous circulation; ACS, acute coronary syndrome; AMI, acute myocardial infarction; HF, heart failure; HCMP, hypertrophic cardiomyopathy; DCMP, dilated cardiomyopathy.

**Table 5:** **Comparison of post-ECPR care according to the neurologic outcome in the mECPR group (n = 52)**

	**CPC 1, 2 (n = 8)**	**CPC 3 to 5 (n = 44)**	***P*** **-value**
ROSB after ECPR	8 (100)	34 (81.0)	0.328
MAP ≥60 mmHg within 2 hrs after ECPR, n (%)	8 (100)	25 (56.8)	0.021
Number of infused vasopressor/inotropics, n	2 (1 to 3)	2 (1 to 2)	0.945
Inotropic equivalent, μg/kg/min for 2 hrs post ECPR	32 (10 to 74)	59 (15 to 82)	0.180
LVEF after ECPR implantation	33 (18 to 48)	10 (0 to 20)	0.009
Therapeutic hypothermia, n (%)	5 (62.5)	9 (20.5)	0.025
ROSB to target temperature (33 °C), hrs	4 (2 to 6)	2.8 (1.6 to 3.0)	0.222 0.435
Time on target temperature, hrs	24 (21 to 24)	24 (24 to 41)	
CAG in patients with suspected ACS, n (%)	7 in 7 (100)	32 in 37 (86.5)	0.574
CAG findings	n =7	n = 32	0.015
No significant coronary lesion	2 (28.6)	3 (9.4)	
Coronary spasm, n (%)	0 (0)	3 (9.4)	
Coronary occlusion/stenosis, n (%)	5 (71.4)	26 (81.2)	
Diseased coronary artery, n (%)	n = 5	n = 26	0.719
1 vessel	4 (80.0)	19 (73.1)	
2 vessels	1 (20.0)	4 (15.4)	
3 vessels	0 (0)	3 (11.5)	
PCI for coronary occlusion/stenosis	5 (100)	24 (92.3)	1.000
Complications during ECLS, n (%)	0 (0)	16 (36.4)	0.047
Bleeding at access site	-	12 (27.3)	
Lower limb ischemia	-	3 (6.8)	
Intracranial hemorrhage	-	1 (2.3)	
Anterograde reperfusion catheter, n (%)	11 ( 25.0)	2 (25.0)	1.000
Amount of transfused pRBC	5 (3 to 12)	5 (1 to 10)	0.833
Successful weaning form ECLS, n (%)	8 (100)	2 (4.5)	<0.001
Duration of ECLS, hrs	43.6 (29.7 to 92.8)	17.9 (3.7 to 48.7)	0.026

Data are shown as median (IQR) or number (%). CPR, cardiopulmonary resuscitation; ECPR, extracorporeal CPR; ROSB, return of spontaneous heart beat; LVEF, left ventricle ejection fraction; CAG, coronary angiography; ACS, acute coronary syndrome; PCI, percutaneous coronary intervention; ECLS, extracorporeal life support; pRBC, packed red blood cells.

### Upper limitation of CPR duration in the ECPR implantation group for expecting a good neurologic outcome

**Figure 7:**
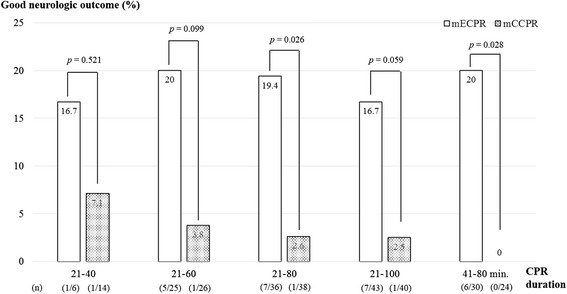
**Comparison of the rate of good neurologic outcomes in the matched extracorporeal cardiopulmonary resuscitation (mECPR) and matched conventional cardiopulmonary resuscitation (mCCPR) groups according to the cardiopulmonary resuscitation (CPR) duration.** CPR duration between 21 to 80 minutes showed that mECPR had a significantly greater rate of good neurologic outcomes (CPC 1, 2). CPC, cerebral performance category.

Because there was no significant difference in CPR duration between good neurologic outcome and poor outcome in the mECPR group (Table 3), we compared neurologic outcomes in the mECPR and mCCPR groups according to the CPR duration (Figure 7). There was no difference in neurological outcome between mECPR and mCCPR when the CPR duration was 21 to 40 minutes. However, mECPR showed a higher rate of good neurological outcomes with 21 to 80 minutes of CPR duration than mCCPR (*P* = 0.026).

## Discussion

This study is one of the few using propensity score for evaluating the impact of ECPR for OHCA. In this study the CCPR group tended to show decreased rates of ROSC, 24-hour survival, survival and good neurological outcome at 3 months post cardiac arrest as the CPR duration increased. In particular, the rate of good neurological outcomes significantly decreased after CPR duration of 21 minutes. The survival analysis for the OHCA patients with CPR duration ≥21 minutes showed a better neurological outcome at 3 months post arrest in the ECPR compared to the CCPR group. Using propensity-score matching, the mECPR group, compared with the mCCPR group showed an improved neurological outcome at 3 months post arrest, despite the survival to discharge rate not showing any difference between groups. In the mECPR group, younger age patients, with witnessed cardiac arrest, who had no asystole as an initial arrest rhythm, were good candidates for ECPR. In addition, early achievement of mean arterial pressure (MAP) ≥60 mmHg, therapeutic hypothermia, and low incidence of ECPR-related complications were significant factors for good neurologic outcome in the mECPR group.

As the CCPR duration becomes longer, optimal perfusion cannot be maintained until the underlying cardiac defect is corrected. Consequently, prolonged CPR is associated with a poor outcome [1,2,15,19,20]. Our study showed that the probability of survival to discharge, especially with a good neurological outcome tends to decrease with longer CPR duration in the CCPR group.

In patients who experience prolonged cardiac arrest with failed CCPR, ECPR can provide an alternative therapeutic option to improve the chances of rapid return of circulation and survival with good neurological outcome [4,14,21,22]. Whether determining an optimal transition time of CPR duration before deploying ECPR results in a better neurological outcome than CCPR remains unknown. Reynolds *et al*. reported that alternative methods should be considered within 16.1 minutes of total CPR duration from chest compression by the EMS provider [1]. However, we have stepped even further and determined that 21 minutes from CPR start by the healthcare provider is an acceptable cutoff time for the CPR duration in predicting a good neurological outcome in the patients who receive only CCPR. In this study, the ECPR group showed a better neurological outcome at 3 months post arrest compared to the CCPR group in patients with a CPR duration ≥21 minutes. This result was similar with the guidelines for indications for the use of ECPR by French medical scientific societies, which recommended that a refractory cardiac arrest prolonged for 30 minutes results in a poor chance of obtaining good neurological outcomes, and ECPR should be considered [14].

ECPR requires many medical resources and has several limitations; primarily, it requires adequate indications for implementation. French medical scientific societies recommended that it is not appropriate to consider extracorporeal life support when CPR has lasted less than 15 minutes [14]. In this study, a good neurological outcome occurred in 31.7% of patients in the CCPR group with a CPR duration <21 minutes. Three patients during our experiment received ECPR due to recurrent arrest after achievement of early ROSC (≥20 minutes) within 20 minutes of CPR duration. However, they have all expired. The importance and advantage of CCPR during a short low-flow time (CPR duration) cannot be replaced by using ECPR.

In this study, there were no differences in the interval from cardiac arrest to CPR start and CPR duration between the group with CPC 1, 2 and the group with CPC 3 to 5 among the mECPR group. Time interval from CPR start to ECPR implantation is also important to outcomes among OHCA patients who required prolonged CPR. Leick *et al*. reported that a door-to-ECPR implantation time of <30 minutes, and not just total CPR duration, had a benefit of 1-month survival in ECPR patients [23]. Fagnoul *et al*. reported that good outcome can be obtained in 15 to 20% of patients, provided that time from arrest to ECPR flow is shorter than 60 minutes [24]. In this study, CPR duration of 21 to 80 minutes resulted in a good neurologic outcome for 19.4% of patients in the mECPR group. If we consider ECPR as an alternative method after 21 minutes of CPR duration, the time of ECPR implantation will be after 40 minutes of CPR duration, due to the time interval from the call for team activation to ECPR implantation. The patients with CPR duration of 41 to 80 minutes showed better neurologic outcome in the mECPR than in the mCCPR group, except the one patient with the longest CPR duration of 120 minutes. This case was previously reported [25]. Our study suggests that OHCA patients who do not respond to conventional CPR given by the healthcare provider within 21 minutes must be considered for ECPR as an alternative method, and earlier implantation of ECPR may be better for expecting a good neurologic outcome, because the rate of good neurological outcomes tend to decrease as the CPR duration increases, even in the mECPR group.

Although this study focused on the CPR duration, many other covariates, including no-flow time, initial arrest rhythm, causes of arrest, refractory arrest or re-arrest, and post-resuscitation care will affect the good neurologic outcome from ECPR. In this study, age was a significant variable for predicting good neurologic outcome in the mECPR group. Among our neurologically intact survivors, the oldest age was 82 years in the CCPR group and 62 years in the ECPR group. Although we could not indicate the absolute age criterion for ECPR in cardiac arrest, younger age might have a more favorable outcome. Previous comorbidity is important to the outcomes of OHCA patients. In this study, there was no difference in comorbidities or SAPS II score between the groups with CPC 1, 2 and CPC 3 to 5 in the mECPR group, because we only considered patients without a serious comorbidity for ECPR.

The AHA recommended ECPR for patients who have a brief no-flow time [8,9]. In this study, no survivor with a good neurologic outcome was reported in ECPR patients with both asystole and unwitnessed cardiac arrest. The no-flow time may be increased among patients with unwitnessed arrest. In addition, asystole may be the last rhythm of the arrest [26]. Serum lactate or arterial pH reflects the severity of tissue hypoxia. In this study, the levels of serum lactate and arterial pH were not different between ECPR and CCPR (Table 1), and between groups with CPC 1, 2 group and CPC 3 to 5 in the mECPR group, respectively (Table 3). We have theorized that the longer time interval from arrest to ED presentation influenced the high levels of serum lactate and development of acidosis in each group.

Post-resuscitation care is also important to the outcomes of OHCA patients. Mollmann *et al*. reported that early invasive treatment after CPR, irrespective of the underlying cause of cardiac arrest, leads to considerably reduced mortality and improved prognosis in patients after OHCA [27]. In our study, many patients undergoing mCCPR expired during CPR or early in the post-ROSC period. The chance of receiving immediate CAG was limited in the mCCPR group. Therefore, the incidence of coronary artery with occlusion or stenosis was lower in the mCCPR than in the mECPR group. However, most patients in the mECPR group immediately received CAG under the support of ECLS when they were suspected of having acute coronary syndrome. ECPR will enhance the opportunity for correction of the cause of cardiac arrest by supporting the essential circulation.

In this study, early achievement of hemodynamic stability after ECPR and application of therapeutic hypothermia were significant factors for good neurologic outcomes among the mECPR group: mECPR patients with CPC 3 to 5 had a high incidence of shock in spite of high-dose infusion of inotropics. We thought that this might be a result of the low incidence of ROSB and low LVEF in mECPR patients with CPC 3 to 5. Hemodynamic instability precluded the application of therapeutic hypothermia. The most common complication during ECPR was bleeding at the access site, possibly related to the technique of catheter insertion. We used methods of sono-guided catheter insertion in the ED and fluoroscope-guided catheter insertion in the catheterization room. In this study, there was no difference in neurologic outcome according to the location of ECPR implantation in the mECPR group.

### Limitations

This study has several limitations that require consideration. First, this was a non-randomized observational cohort study from retrospective analysis in a single center. Pertinent indication criteria of ECPR have not been established and the protocol has been not standardized; it differs according to the emergency medical services (EMS) and in-hospital system. All studies on ECPR, including our study, have different inclusion criteria and methods of intervention and analysis under different healthcare environments. Therefore, the transition time for considering ECPR may be changeable according to the definition of CPR duration or different EMS service system. Our study suggests that the physician who works with ECPR has to find the specific transition time for ECPR that suits their EMS system. Second, the upper limit of CPR duration for ECPR implantation was not clearly revealed in this study. The small cohort study prevented many parameters from achieving statistical significance. It may be necessary to perform a prospective multi-center, randomized controlled study with a clear protocol to determine the precise difference between neurological outcomes with ECPR and CCPR. Third, the standard CPR guideline has been changed during the study period. The change of guideline might affect the CPR performance in this study group. Before 2010 therapeutic hypothermia was not frequently used. Indeed, we avoided the induction of hypothermia, which could improve neurologic recovery, in ECPR patients during 2006 to 2008, because of concerns about hypothermia inducing unstable vital signs and tendency for bleeding. After the introduction of the 2010 AHA guideline, therapeutic hypothermia was more frequently applied post resuscitation in CCPR and ECPR patients [4]. Fourth, we compared the outcomes of the mECPR and the mCCPR groups under identical inclusion criteria after matching. However, several variables might not be identically matched and unmeasured bias may remain despite the use of propensity-score-matching. In this study, the incidence of TH in the mCCPR group was lower compared with that in the mECPR group. However, the rate of TH in patients with ROSC or ROSB was high with mCCPR compared to mECPR.

## Conclusions

A good neurological outcome declined with prolonged CPR duration. ECPR should be considered as an alternative method for attaining good neurological outcomes in OHCA patients who require prolonged CPR, especially CPR ≥21 minutes. Younger patients, with witnessed cardiac arrest without initial asystole were good candidates for expecting good neurologic outcome from ECPR. After implantation of ECPR, early hemodynamic stabilization, prevention of ECPR-related complications, and application of therapeutic hypothermia may improve the neurologic outcome.

## Key messages

- Good neurologic outcome can be expected when we transit from CCPR to ECPR, especially in patients requiring prolonged CPR, particularly for CPR duration of ≥21 minutes
- Younger age patients with witnessed cardiac arrest without initial asystole were good candidates for ECPR in OHCA patients who required prolonged CPR
- After implantation of ECPR, early achievement of hemodynamic stability, prevention of ECPR-related complication, and therapeutic hypothermia improved the neurologic outcome

## Abbreviations

ACS: acute coronary syndrome
AHA: American Heart Association
CAG: coronary angiography
CCPR: conventional cardiopulmonary resuscitation
CPC: cerebral performance category
CPR: cardiopulmonary resuscitation
DNAR: do not attempt resuscitation
ECLS: extracorporeal life support
ECMO: extracorporeal membrane oxygenation
ECPR: extracorporeal cardiopulmonary resuscitation
ED: emergency department
KUMC: Korea University Medical Center
LOS: length of stay
mCCPR: matched conventional cardiopulmonary resuscitation
mECPR: matched extracorporeal cardiopulmonary resuscitation
OHCA: out-of-hospital cardiac arrest
PCI: percutaneous coronary intervention
ROSB: return of spontaneous heart beating
ROSC: return of spontaneous circulation
